# Bovine Lactoferrin Enhances Toll-like Receptor 7 Response in Plasmacytoid Dendritic Cells and Modulates Cellular Immunity

**DOI:** 10.3390/ijms252413369

**Published:** 2024-12-13

**Authors:** Takumi Yago, Asuka Tada, Shutaro Kubo, Hirotsugu Oda, Sadahiro Iwabuchi, Miyuki Tanaka, Shinichi Hashimoto

**Affiliations:** 1 R&D Division, Morinaga Milk Industry Co., Ltd., Zama 252-8583, Japan; 2 International Division, Morinaga Milk Industry Co., Ltd., Minato 105-7122, Japan; 3 Department of Molecular Pathophysiology, Wakayama Medical University, Wakayama 641-8509, Japan

**Keywords:** bovine lactoferrin, plasmacytoid dendritic cells, peripheral blood mononuclear cells, immune cells, interferon, toll-like receptor 7

## Abstract

Plasmacytoid dendritic cells (pDCs) express Toll-like receptor 7 (TLR7) in the endosomes, recognize viral single-stranded RNA (ssRNA), and produce significant amounts of interferon (IFN)-α. Bovine lactoferrin (LF) enhances the response of IFN regulatory factors followed by the activation of IFN-sensitive response elements located in the promoter regions of the *IFN-α* gene and IFN-stimulated genes in the TLR7 reporter THP-1 cells in the presence of R-848, a TLR7 agonist. In ex vivo experiments using human peripheral blood mononuclear cells, LF enhances IFN-α levels in the supernatant in the presence of R-848. Additionally, it increases the expression of IFN-α, human leukocyte antigen (HLA)-DR, and CD86 in pDCs; HLA-DR and CD86 in myeloid dendritic cells; CD69 in CD56 dim natural killer and T killer cells; and IFN-γ in T helper type 1 and B cells in the presence of R-848. The inhibition of phagocytosis or neutralization of nucleolin, a receptor of LF, suppresses LF incorporation into pDCs. These results suggest that pDCs incorporate LF through phagocytosis or nucleolin-mediated endocytosis, and LF enhances TLR7 response in the endosome and subsequent IFN signaling pathway and activates innate and adaptive immune cells. We anticipate that LF modulates antiviral immunity against environmental ssRNA viruses and contributes to homeostasis.

## 1. Introduction

Rhinovirus [1], coronavirus [2], respiratory syncytial virus [3], parainfluenza virus [4], influenza virus [5], and enterovirus [6] are single-stranded RNA (ssRNA) viruses known to cause cold-like symptoms. Plasmacytoid dendritic cells (pDCs) express the Toll-like receptor (TLR) 7, recognize viral ssRNA, and produce substantial amounts of interferon (IFN)-α. IFN-α subsequently upregulates hundreds of IFN-stimulated genes and activates myeloid dendritic cells (mDCs), natural killer (NK) cells, helper and killer T cells, and B cells. pDCs express major histocompatibility complex (MHC) class II molecules, such as human leukocyte antigen (HLA)-DR, and co-stimulatory molecules, such as CD86, and present viral antigens to naive T cells [7,8]. Therefore, pDCs are key players in the modulation of innate and adaptive immunity in response to environmental viruses.

Lactoferrin (LF) is an iron-binding glycoprotein present in plasma and exocrine fluids, such as milk, tears, nasal and vaginal secretions, and saliva. Ocular, nasal, vaginal, and oral mucosa covered by these exocrine fluids are located near the body surface, and its surface is constantly exposed to environmental antigens. Therefore, LF in the exocrine fluids may modulate the immune response to the environmental antigens and contribute to homeostasis. LF isolated from bovine milk is widely used as a functional food ingredient [9] and may be useful in immune function maintenance. Oral administration of LF induces the production of IFN-α/β in Peyer’s patches and activates NK cells in mice [10]. It also activates helper and killer T cells and enhances the production of immunoglobulin A in B cells in Peyer’s patches and lamina propria in mice [11]. However, whether LF modulates the activity of pDCs is not examined in these studies.

LF does not induce IFN-α production from peripheral blood mononuclear cells (PBMCs) in the absence of ssRNA, but LF induces its production and enhances the expression of IFN-α, HLA-DR, and CD86 in pDCs in PBMCs in the presence of ssRNA ex vivo [12]. Additionally, the intake of LF (200 mg/day) enhances the expression of IFN-α, HLA-DR, and CD86 of pDCs in PBMCs of healthy adults [13,14]. As humans are constantly exposed to environmental antigens, including ssRNA viruses, endogenous or exogenous LF may synergistically enhance the activity of pDCs in PBMCs with foreign TLR7 ligands. However, there are limited data on how LF affects pDCs and whether it also enhances the activity of other co-existing immune cells, such as mDCs, NK cells, helper and killer T cells, and B cells in the complex system of PBMCs. 

The purpose of the present study is to clarify how LF affects pDCs and whether it affects immune cells other than pDCs in PBMCs under pDC-activated conditions. Therefore, we assessed the effects of LF on TLR7 reporter THP-1 cells, pDCs, and other immune cells in PBMCs, in the presence of a synthetic TLR7 agonist.

## 2. Results

### 2.1. Response of Interferon (IFN) Regulatory Factor (IRF) Followed by Activation of IFN-Sensitive Response Element (ISRE) in Toll-like Receptor (TLR) 7 Reporter THP-1 Cells

**Figure 1:**
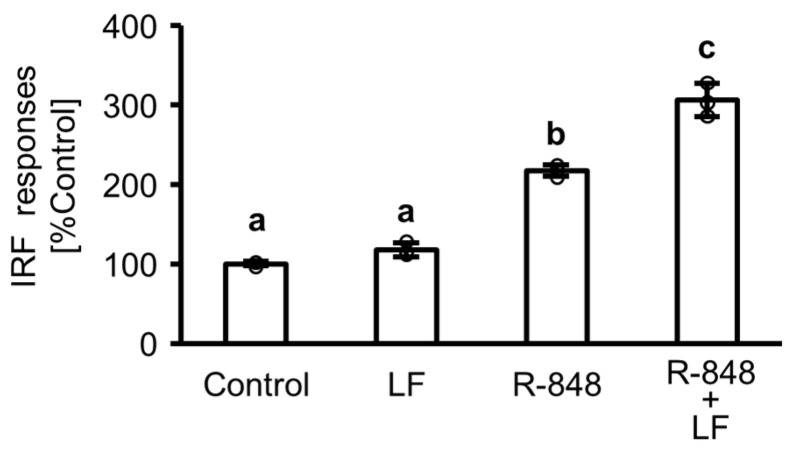
Interferon (IFN) regulatory factor (IRF) response followed by activation of IFN-sensitive response element (ISRE) in Toll-like receptor 7 reporter THP-1 cells. After 6 h of incubation in the presence or absence of 100 µg/mL lactoferrin (LF) and 10 µg/mL R-848, IRF response followed by ISRE activation was assessed using a luciferase reporter assay. Values are presented as the mean and standard deviation (SD); Open circles represent individual values. n = 3. Different letters above the bars (a, b, c) indicate significant differences among treatments (*p* < 0.05).

Figure 1 illustrates the IFN regulatory factor (IRF) response followed by IFN-sensitive response element (ISRE) activation in response to TLR7 stimulation in TLR7 reporter THP-1 cells following incubation with LF and R-848, a TLR7 agonist. In the absence of R-848, LF addition exhibited no effect on IRF response. In contrast, R-848 addition significantly increased the IRF response, and the combination of LF and R-848 further enhanced this effect. In the subsequent experiments, we assessed the effects of LF in the presence of R-848.

### 2.2. IFN-α Production from Peripheral Blood Mononuclear Cells (PBMCs) Following Lactoferrin (LF) Treatment

**Figure 2:**
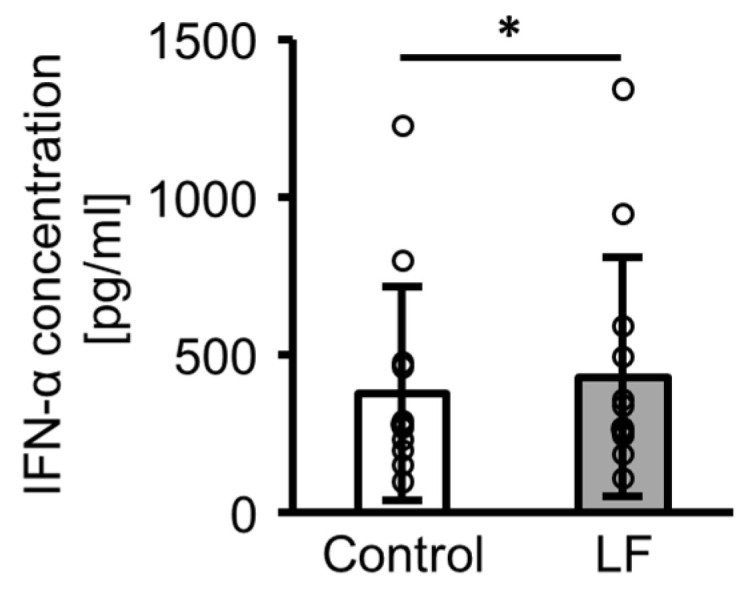
IFN-α concentration in peripheral blood mononuclear cell (PBMC) culture supernatants. After 24 h incubation of PBMCs with 10 µg/mL R-848 (control) or 10 µg/mL R-848 and 100 µg/mL LF, IFN-α concentration in culture supernatants was measured using an ELISA kit. White bars represent the control group, and gray bars represent the LF-treated group. Values are presented as the mean and SD; Open circles represent individual values. n = 11. * Significantly different from the control group (*p* < 0.05). LF, lactoferrin.

Figure 2 illustrates the IFN-α concentration following incubation with LF in the PBMC culture supernatants. In the presence of R-848, LF treatment significantly enhanced the IFN-α concentration. 

### 2.3. Plasmacytoid Dendritic Cell (pDC) Activity in PBMCs Following LF Treatment

**Figure 3:**
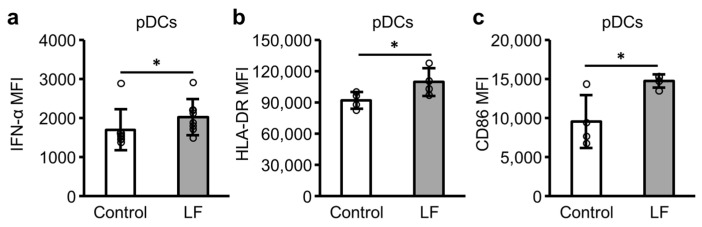
Intracellular IFN-α, cell surface human leukocyte antigen (HLA)-DR, and cell surface CD86 expression levels in plasmacytoid dendritic cells (pDCs). After 20–24 h incubation of peripheral blood mononuclear cells with 10 µg/mL R-848 (control) or 10 µg/mL R-848 and 100 µg/mL LF, the expression levels of pDC activity markers, (**a**) intracellular IFN-α (n = 7), (**b**) cell surface HLA-DR (n = 4), and (**c**) cell surface CD86 (n = 4) were measured using flow cytometry. CD123+CD304+ cells are defined as pDCs. White bars represent the control group, and gray bars represent the LF-treated group. Values are presented as the mean and SD. Open circles represent individual values. * Significantly different from the control group (*p* < 0.05). MFI, geometric mean fluorescence intensity. LF, lactoferrin.

Figure 3 illustrates the intracellular IFN-α, cell surface HLA-DR, and cell surface CD86 expression levels in pDCs following the incubation of PBMCs with LF. In the presence of R-848, the addition of LF significantly enhanced the expression levels of intracellular IFN-α (Figure 3a) and the cell surface markers HLA-DR and CD86 (Figure 3b,c) in pDCs.

### 2.4. Immune Cell Activity Following LF Treatment

**Figure 4:**
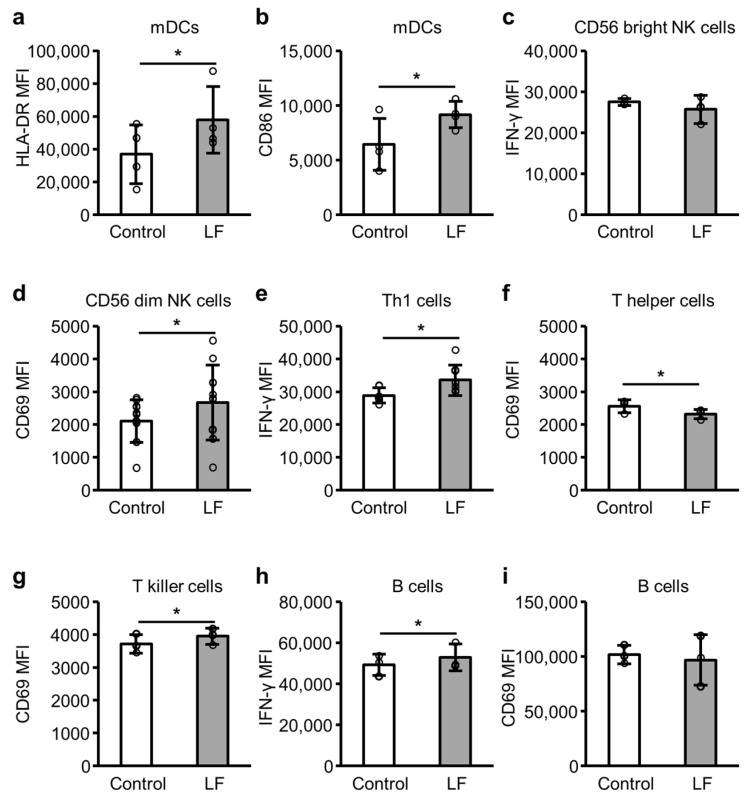
Expression of activation markers in the immune cells. After 6–24 h incubation of peripheral blood mononuclear cells with 10 µg/mL R-848 (control) or 10 µg/mL R-848 and 100 µg/mL LF, the expression levels of immune cell activity markers were measured using flow cytometry. (**a**) Cell surface HLA-DR and (**b**) CD86 in myeloid dendritic cells (mDCs, CD11c+CD123− cells) (n = 4). (**c**) Intracellular IFN-γ in CD56 bright natural killer (NK) cells (CD3−CD56 bright cells) (n = 3). (**d**) Cell surface CD69 in CD56 dim NK cells (CD3−CD16+CD56 dim cells) (n = 11). (**e**) Intracellular IFN-γ in T helper type 1 (Th1) cells (CD3+CD4+CD183+ cells) (n = 7). Cell surface CD69 in (**f**) T helper cells (CD3+CD4+ cells) (n = 3) and (**g**) T killer cells (CD3+CD8+ cells) (n = 3). (**h**) Intracellular IFN-γ in B cells (CD19+ cells) (n = 7). (**i**) Cell surface CD69 in B cells (CD19+ cells) (n = 3). White bars represent the control group, and gray bars represent the LF-treated group. Values are presented as the mean and SD. Open circles represent individual values. * Significantly different from the control group (*p* < 0.05). MFI, geometric mean fluorescence intensity; LF, lactoferrin.

Figure 4 illustrates the expression levels of activation markers in immune cells. In the presence of R-848, the addition of LF significantly enhanced the expression levels of cell surface HLA-DR and CD86 in mDCs (Figure 4a,b), cell surface CD69 in CD56 dim NK (Figure 4d) and T killer (Figure 4g) cells, and intracellular IFN-γ in T helper type 1 (Th1) (Figure 4e) and B (Figure 4h) cells. In contrast, the addition of LF reduced cell surface CD69 expression levels in T helper cells (Figure 4f) and did not affect the expression levels of intracellular IFN-γ in CD56 bright NK cells (Figure 4c) and cell surface CD69 in B cells (Figure 4i).

### 2.5. The Cellular Uptake of LF in pDCs

**Figure 5:**
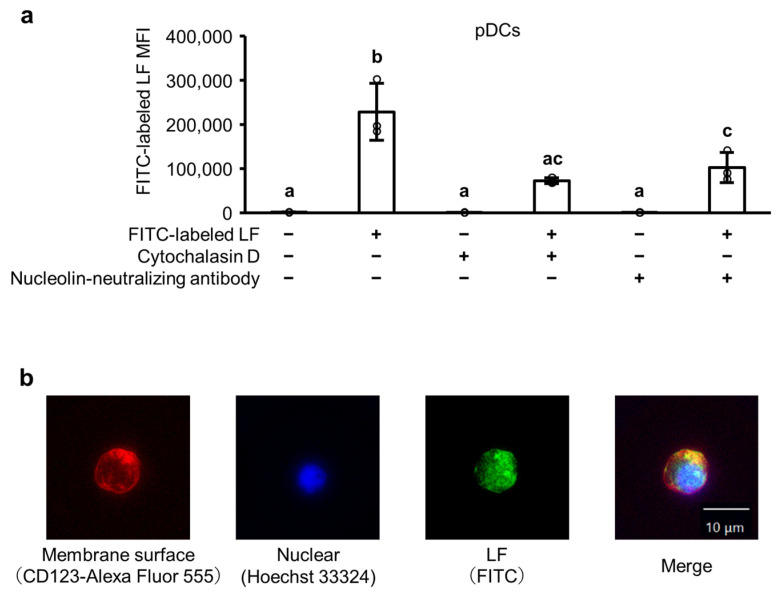
Incorporation of LF into pDCs. (**a**) Fluorescein isothiocyanate (FITC) fluorescence signals of pDCs, as measured through flow cytometry after 24 h incubation of peripheral blood mononuclear cells with 10 µg/mL R-848 and 100 µg/mL FITC-labeled LF, in the presence or absence of 1 µM cytochalasin D or 5 µg/mL nucleolin-neutralizing antibody. Values are presented as the mean and SD; Open circles represent individual values. n = 3. Different letters above the bars (a, b, c) indicate significant differences among treatments (*p* < 0.05). (**b**) Images captured of pDCs, as measured through fluorescence microscopy, after 24 h incubation of isolated pDCs with 10 µg/mL R-848 and 100 µg/mL FITC-labeled LF. Green: FITC-labeled LF; red: pDC membrane surface (CD123); and blue: nuclear staining with Hoechst 33324. Magnification: ×100; scale bar: 10 μm. LF, lactoferrin; pDCs, plasmacytoid dendritic cells.

Figure 5a illustrates the fluorescein isothiocyanate (FITC) fluorescence signals of pDCs in PBMCs after incubation with FITC-labeled LF in the absence or presence of cytochalasin D and a nucleolin-neutralizing antibody. The addition of FITC-labeled LF significantly enhanced the FITC fluorescence signals of pDCs, whereas treatment with cytochalasin D or a nucleolin-neutralizing antibody significantly reduced the FITC fluorescence signal. Figure 5b illustrates the fluorescence microscopy images of pDCs incubated with FITC-labeled LF. The accumulation of FITC-labeled LF in the cytoplasm of pDCs was observed within 24 h. 

## 3. Discussion

The purpose of this study was to clarify how LF affects pDCs, and whether LF affects other co-existing immune cells in PBMCs under pDC-activated conditions. We demonstrated that LF was incorporated into pDCs through phagocytosis or nucleolin-mediated endocytosis, enhanced TLR7 response in the endosomes, upregulated the IFN signaling pathway, and activated various innate and adaptive immune cells.

TLR7 in the endosomes of immune cells recognizes viral ssRNA and induces IFN signaling [15]. To measure IRF response followed by ISRE activation in response to TLR7 stimulation, we used TLR7 reporter THP-1 cells containing an ISRE-driven luciferase reporter gene stimulated with LF and R-848, a TLR7 agonist. ISRE is located in the promoter regions of the *IFN-α* gene and IFN-stimulated genes, with gene expression induced through IRF binding. In the absence of R-848, LF did not induce IRF response. In contrast, in the presence of R-848, IRF response was induced, and LF enhanced its response. Therefore, LF may upregulate IRF response followed by the expression of the *IFN-α* gene and IFN-stimulated genes in the presence of a TLR7 agonist, such as viral ssRNA.

Similarly, in the absence of R-848, PBMCs did not produce IFN-α and LF did not induce its production [12]. In contrast, in the presence of R-848, PBMCs produced IFN-α and LF enhanced its production. All PBMC donors were healthy, without autoimmune diseases or infections. Chronic IFN-α production is a risk factor for autoimmune diseases, such as systemic lupus erythematosus [16]; therefore, it is reasonable that neither PBMCs produce IFN-α nor LF induces its production unless TLR7 signals an emergency. LF enhances IFN-α production in the presence of ssRNA, a TLR7/8 agonist, or poly (I:C), a TLR3 agonist [12,17]. Poly (I:C) is a double-stranded RNA observed during viral replication. Therefore, LF may synergistically induce IFN-α production with viral single- or double-stranded RNA. In contrast, LF inhibits IFN-α production induced by bacterial CpG DNA, a TLR9 agonist [18], possibly suppressing the excess immune response against commensal bacteria. Notably, the immunomodulatory effects of LF on the viral and bacterial genomes are different.

pDCs are the primary producers of IFN-α in PBMCs [7]; therefore, we assessed the effect of LF on pDCs in PBMCs. As anticipated, LF enhanced the intracellular expression of IFN-α and cell surface expression of HLA-DR and CD86 on pDCs in PBMCs in the presence of R-848. Therefore, LF likely activates pDCs in the presence of a TLR7 agonist, such as viral ssRNA in the environment.

IFN-α production and antigen presentation by pDCs through MHC class II and co-stimulatory molecules may contribute to the activation of innate and adaptive immunity; therefore, we also assessed the effects of LF on other immune cells, such as mDCs, NK cells, T cells, and B cells in PBMCs. LF enhanced the expression of HLA-DR and CD86 in mDCs, CD69 in CD56 dim NK and T killer cells, and IFN-γ in T helper and B cells. IFN-α enhances the cell surface expression of HLA-DR and CD86 on mDCs [19], upregulates CD69 cell surface expression on NK cells [20], increases IFN-γ-producing Th1 cells [21], primes naive B cells for IFN-γ production [22], and upregulates CD69 expression on T killer cells [23]. Therefore, the increase in pDC-derived IFN-α by LF possibly contributed to the activation of mDCs, NK cells, T killer cells, Th1 cells, and B cells. We have to examine whether the depletion of pDCs from PBMCs or neutralization of IFN-α reduces the effect of LF to make this hypothesis more reliable. pDCs as well as B cells and monocytes express TLR7 [24]; therefore, LF may directly affect immune cells other than pDCs, and these immune cells interact with each other. The effects of LF on individual immune cells separated from PBMCs are of interest. LF did not enhance the expression of IFN-γ in CD56 bright NK cells, and CD69 in T helper and B cells in PBMCs in the presence of R-848. CD56 dim NK cells are more cytotoxic, whereas CD56 bright NK cells can produce abundant cytokines, such as IFN-γ [25]. Therefore, LF may preferentially activate the cytotoxic NK cells. CD69 serves as a very early activation marker [26], which can make assessments based on the time point challenging in the case of T helper and B cells. Nevertheless, to the best of our knowledge, this is the first report that LF simultaneously activates pDCs, mDCs, NK cells, T killer cells, Th1 cells, and B cells in the complex system of PBMCs in the presence of the TLR7 ligand. Although we have to reconfirm these results using not synthetic TLR7 ligands but real ssRNA viruses, we anticipate that LF modulates antiviral immunity against environmental ssRNA viruses and contributes to homeostasis.

pDCs are a possible significant target for antiviral immunomodulation by LF; therefore, we assessed how LF attaches to or enters pDCs using fluorescent-labeled LF. pDCs are phagocytes and phagocytose environmental antigens [27]; therefore, LF may be incorporated into pDCs using this route. As anticipated, cytochalasin D—an inhibitor of actin-dependent phagocytosis and macropinocytosis—inhibited the incorporation of fluorescent-labeled LF into pDCs to a certain extent. pDCs express nucleolin [28], low-density lipoprotein receptor-related protein 1 (LRP1) [29], and C-X-C chemokine receptor type 4 (CXCR4) [30] on their cell surfaces, which are reported to function as LF receptors in other cells [31]. Therefore, LF may be incorporated into pDCs using a receptor-specific route. Neutralizing antibodies against nucleolin inhibited the incorporation of fluorescent-labeled LF into pDCs. In contrast, the neutralization of LRP1 or CXCR4 did not alter the incorporation in our preliminary experiments. LF is reported to co-localize with cell surface nucleolin and is endocytosed into the endosome through a clathrin-dependent pathway in other cells [32]. Nucleolin shuttles nucleic acid TLR agonists, such as poly (I:C) and CpG DNA, from the cell surface to TLR3 and TLR9 within the endosomes of mDCs and pDCs [28]. Nucleolin expressed on the plasma membrane acts as a co-receptor for the attachment and entry of multiple ssRNA viruses, such as respiratory syncytial virus, enterovirus A 71, human immunodeficiency virus type 1, various influenza A virus subtypes, and human parainfluenza virus type 3 [33]. These findings suggest that LF, viruses, and viral ssRNA in the environment are phagocytosed or endocytosed, and LF and viral ssRNA are transferred to TLR7 within the endosomes of pDCs. LF and viral ssRNA are positively and negatively charged, respectively; therefore, the cation–anion interaction between LF and viral ssRNA may modulate the recognition of viral ssRNA by TLR7 within the endosomes of pDCs.

Considering our findings, endogenous LF in exocrine fluids, such as tears, nasal and vaginal secretions, and saliva may modulate the response of immune cells against environmental ssRNA viruses that invade the mucosal surface. Orally ingested exogenous LF may be absorbed through the sublingual route [34] or survive stomach conditions [35,36] and be absorbed into the intestine through the lymphatic pathway [37]. Subsequently, it may act on immune cells, including pDCs in the peripheral blood. However, immune cells also exist in the lymphoid tissues of the digestive tract such as palatine tonsils and mesenteric lymph nodes [38,39], where LF can reach more easily than peripheral blood. Therefore, orally ingested LF may also modulate the response of immune cells in the digestive tract [10,11]. Immune cells in the peripheral blood and digestive tract may exhibit different characteristics [38]; therefore, further studies to assess the effects of LF on immune cells, including pDCs in the digestive tract are required. Orally ingested LF activates pDCs within PBMCs [13,14]; therefore, these studies will promote the understanding of LF bioactivity in vivo and the development of functional foods containing LF.

## 4. Materials and Methods

### 4.1. Ethical Approval

This study adhered to the current revision of the Declaration of Helsinki (2013) and Ethical Guidelines for Medical and Health Research Involving Human Subjects (2015). The research protocol and informed consent form were approved by the Institutional Review Board (IRB) of Wakayama Medical University, Japan (Approval No. 3062).

### 4.2. Materials

In this study, we used LF with a purity of 97.2% and an iron content of 17.8 mg/100 g purified from skimmed milk (Morinaga Milk Industry, Tokyo, Japan) in PBMC culture, and LF with a purity of 95% (#122-06811) was obtained from Fujifilm Wako (Tokyo, Japan) in an IRF response assay.

FITC-labeled LF was prepared by dissolving 1000 mg of LF and 10 mg of FITC-I (Dojindo Laboratories, Kumamoto, Japan) in 0.1 M phosphate buffer (pH 8.0). The mixture was gently shaken for 2.5 h at room temperature in the dark, followed by centrifugation (10,000 × g, 5 min) to remove insoluble materials. The supernatant was dialyzed using a Spectra/Por 7 membrane (MWCO = 10,000; Funakoshi, Tokyo, Japan) to remove unreacted FITC and phosphate buffer components. The FITC-labeled LF was recovered by freezing at −35 °C and was subsequently lyophilized.

R-848, a TLR7/8 ligand obtained from InvivoGen (San Diego, CA, USA), has potent antiviral activity and is used as an inducer of IFN-α production in pDCs within PBMCs through the TLR7 signaling pathway. It was stored at −20 °C until use.

LF, FITC-labeled LF, and R-848 were aseptically dissolved in sterilized phosphate-buffered saline (PBS; Fujifilm Wako) and filtered through a 0.22 µm sterile filter membrane (Millipore, Burlington, MA, USA) before use in cell assays.

### 4.3. IRF Response Assay

The effect of LF on IRF response was assessed using human TLR8 knockout and TLR7-overexpressing THP-1 monocytes containing dual IRF and NF-kB activity reporters (TLR7 reporter THP-1 cells; InvivoGen). Thse TLR7 reporter THP-1 cell culture and IRF response assay followed the manufacturer’s protocol. TLR7 reporter THP-1 cells were cultured in a Roswell Park Memorial Institute 1640 medium (RPMI 1640; Sigma-Aldrich, St. Louis, MO, USA) supplemented with 2 mM L-glutamine, 10% fetal bovine serum (Gibco, Grand Island, NY, USA), 100 U/mL penicillin, 100 μg/mL streptomycin (Fujifilm Wako), 100 μg/mL normocin, 100 μg/mL zeocin, and 10 μg/mL blasticidin (InvivoGen). The cells were maintained at 37 °C in a 5% CO_2_ incubator. For the IRF response assay, TLR7 reporter THP-1 cells were seeded at a density of 1 × 10^5^ cells/well in 96-well plates in RPMI 1640 (without normocin, zeocin, and blasticidin) and incubated in the absence or presence of 100 µg/mL LF and 10 µg/mL R-848 for 6 h at 37 °C in 5% CO_2_. The treatment concentration and incubation time for LF and R-848 were determined using the results of the preliminary experiments. We did not use a positive control because the manufacturer’s protocol indicated that R-848 works in this system. We confirmed that R-848 enhances the IRF response in a concentration-dependent manner in preliminary experiments. We used PBS as the negative control and confirmed that PBS does not affect the IRF response. Following incubation, 20 μL of supernatant was mixed with 50 μL of luciferase reagent, QUANTI-Luc™ 4 Reagent (InvivoGen), in a white 96-well opaque plate, and luminescence was measured using a luminometer (Corona Electric, Ibaraki, Japan) to assess the IRF response. Before measurements, the instrument was initialized and self-calibrated according to the manufacturer’s instructions. TLR7 reporter THP-1 cells were seeded in triplicate wells per condition and used for evaluation. Additionally, the reproducibility of the results was confirmed through repeated experiments.

### 4.4. Preparation of PBMCs

Eligible donors of peripheral blood were healthy adults aged 18 years or older; the principal investigator explained the details of this study and voluntary written informed consent was obtained. Those who met any of the following criteria were excluded from participation: pregnant individuals, and those deemed unsuitable for the study by the principal investigator. Personal data other than information in Table 1 were not disclosed to researchers. Peripheral blood was collected from 16 healthy adults in Table 1 using Vacutainer CPT tubes (BD Biosciences, Franklin Lakes, NJ, USA) after obtaining informed consent. No specific restrictions were placed on the donor’s activities prior to blood collection. Following peripheral blood collection, the tubes were centrifuged (1500× *g*, 20 min, 25 °C), and the fraction containing mononuclear cells was collected and washed with PBS. To remove residual red blood cells, hemolysis was performed using an ammonium chloride solution (STEMCELL Technologies, Vancouver, BC, Canada) according to the manufacturer’s protocol. Ammonium chloride solution was added to the cell suspension at a volume/volume ratio of 1:9, and the mixture was placed on ice for 10 min. Following hemolysis, the cells were washed again with PBS and used as PBMCs for the experiment. PBMCs were maintained on ice until the start of the experiment.

### 4.5. PBMC Culture

PBMCs were seeded at a density of 5 × 10^5^ cells/well in 24-well plates and cultured in RPMI 1640 supplemented with 2 mM L-glutamine, 5% human AB serum (Sigma-Aldrich), 100 U/mL of penicillin, and 100 μg/mL of streptomycin at 37 °C in a 5% CO_2_ incubator. PBMCs from at least three different donors were used for each experiment. The number of donors is described in each figure. PBMCs were seeded in one well per condition. The experimental conditions are as follows:

Experiment 1: To assess the effect of LF on IFN-α production in PBMCs, PBMCs were cultured with 10 µg/mL R-848 in the presence or absence of 100 µg/mL LF for 24 h. Following incubation, PBMC culture supernatants were collected, and IFN-α concentration was measured using a Human IFN-alpha All Subtype ELISA Kit (PBL Assay Science, Piscataway, NJ, USA), following the manufacturer’s protocol.

Experiment 2: To assess the effect of LF on immune cells in PBMCs, PBMCs were cultured with 10 µg/mL R-848 in the presence or absence of 100 µg/mL LF. The PBMCs used to assess the expression levels of cell surface markers were cultured for 6–24 h, whereas those used for assessing intracellular markers were cultured for 20 h and treated with 0.5 μL of a protein transport inhibitor, brefeldin A (BD Biosciences), to accumulate cytokines produced in the cells at the 8 h time point.

Experiment 3: To assess the cellular uptake of LF in pDCs within PBMCs, PBMCs were precultured with 1 µM cytochalasin D (Sigma-Aldrich), a phagocytosis inhibitor [40], or 5 µg/mL neutralizing antibody against nucleolin (Santa Cruz Biotechnology, Dallas, TX, USA) for 1 h [41]. Then, 100 µg/mL FITC-labeled LF and 10 µg/mL R-848 were added and incubated for an additional 24 h, to assess the involvement of LF in endocytosis.

### 4.6. Flow Cytometry

After incubating the PBMCs for 6–24 h, they were washed with PBS and stained with Horizon Fixable Viability Stain 780 (BD Biosciences) for 15 min at room temperature in the dark to exclude dead cells. The cells were washed with Stain Buffer (BD Biosciences) and treated with Human BD Fc Block (BD Biosciences) for 10 min on ice to prevent the false-positive binding of antibodies. Subsequently, the cells were stained with fluorescent-conjugated antibodies, CD3-PreCP-Cy5.5, CD4-FITC, CD8-PE-Cy7, CD11c-PreCP-Cy5.5, CD16-FITC, CD19-PE-Cy7, CD69-PE, CD86-APC, CD123-PE-Cy7, CD183-APC, CD304-BB515, and HLA-DR-PE, or their isotype controls (BD Biosciences) for 30 min on ice in the dark. The stained cells were washed with staining buffer and fixed with 4% paraformaldehyde (Muto Pure Chemical, Tokyo, Japan) for 20 min on ice in the dark. Fixed cells were washed with Stain Buffer and analyzed using a CytoFLEX (Beckman Coulter, Brea, CA, USA). Standardization was performed using QC beads (Beckman Coulter) according to the manufacturer’s instructions before measurement. To assess the expression levels of intracellular markers (IFN-α and IFN-γ), cells were further treated with Perm/Wash Buffer (BD Biosciences) for 15 min on ice in the dark to permeabilize them. The permeabilized cells were stained with IFN-α-Alexa Fluor 647 or IFN-γ-PE for 1 h in the dark. Following staining, the labeled cells were washed with the Perm/Wash Buffer and analyzed using a CytoFLEX (Beckman Coulter). Data analysis was performed using the CytExpert 2.4 software (Beckman Coulter). In the flow cytometry analysis, dead cells were excluded, and single mononuclear cells were selected from the total PBMCs which were gated on a forward scatter (FSC)/side scatter (SSC) plot. Lymphocyte subsets were defined as follows: pDCs = CD123+CD304+; mDCs = CD11c+CD123−; T killer cells = CD3+CD8+; T helper cells = CD3+CD4; Th1 cells = CD3+CD4+CD183; B cells = CD19+; CD56 dim NK cells = CD3−CD16+CD56dim; and CD56 bright NK cells = CD3−CD56 bright (Figure S1). The expression levels of activation markers in the immune cells were calculated as geometric mean fluorescence intensity. Color compensation was used to eliminate false-positive fluorescence in the channels.

### 4.7. Isolated pDC Culture and Fluorescence Microscopy Assay

The cellular uptake of LF in pDCs was analyzed using human peripheral blood pDCs (isolated pDCs; STEMCELL Technologies). Isolated pDCs were seeded at a density of 5 × 10^4^ cells/well in 96-well plates and cultured with 100 µg/mL FITC-labeled LF and 10 µg/mL R-848 in RPMI 1640, supplemented with 2 mM L-glutamine, 5% human AB serum, 100 U/mL of penicillin, and 100 μg/mL of streptomycin at 37 °C in 5% CO_2_ for 24 h. Following incubation, isolated pDCs were washed with Stain Buffer and treated with the Human BD Fc Block for 10 min. Subsequently, the pDC nuclei were stained with Hoechst 33324 (Dojindo Laboratories), whereas the membrane surface was stained with CD123-Alexa Fluor 555 (AAT Bioquest, Pleasanton, CA, USA) for 30 min on ice in the dark. The stained cells were imaged using an all-in-one fluorescence microscope (BZ-X800L; KEYENCE, Tokyo, Japan) at ×100 magnification.

### 4.8. Statistical Analysis

All values are expressed as the mean ± standard deviation. Differences between the two groups were analyzed using a paired Student’s *t*-test. Tukey’s test was used to compare the differences among more than three groups. Statistical significance was set at *p* < 0.05.

## 5. Conclusions

We have revealed that LF is incorporated into pDCs through phagocytosis or nucleolin-mediated endocytosis, enhances TLR7 response and the subsequent IFN signaling pathway, and activates innate and adaptive immune cells. Our future challenge will be to examine the interaction between LF and immune cells other than pDCs and the interaction between immune cells; to consider the in vivo kinetics of LF and different characteristics of immune cells between peripheral blood and the digestive tract; and to use not synthetic TLR7 ligands but real ssRNA viruses.

## Figures and Tables

**Table 1:** Characteristics of PBMC donors.

Age (Years)	Total	Men	Women
20–29	3	1	2
30–39	8	5	3
40–49	4	3	1
50–59	1	1	0
Total	16	10	6

## Data Availability

The data that support the findings of this study are available from the corresponding author upon reasonable request.

## Supplementary Materials

The following supporting information can be downloaded at https://www.mdpi.com/article/10.3390/ijms252413369/s1.
