# Supplementary material for: Bovine Lactoferrin Enhances Toll-like Receptor 7 Response in Plasmacytoid Dendritic Cells and Modulates Cellular Immunity
Source: Int J Mol Sci. 2024 Dec 13;25(24):13369. doi: 10.3390/ijms252413369 (PMC11676446; doi:10.3390/ijms252413369)
Supplement: Supplementary file 1 [file ijms-25-13369-s001.zip › ijms-3314179-supplementary.pdf]

Supplementary Materials for Bovine lactoferrin enhances Toll-like receptor 7 response in plasmacytoid dendritic cells and modulates cellular immunity

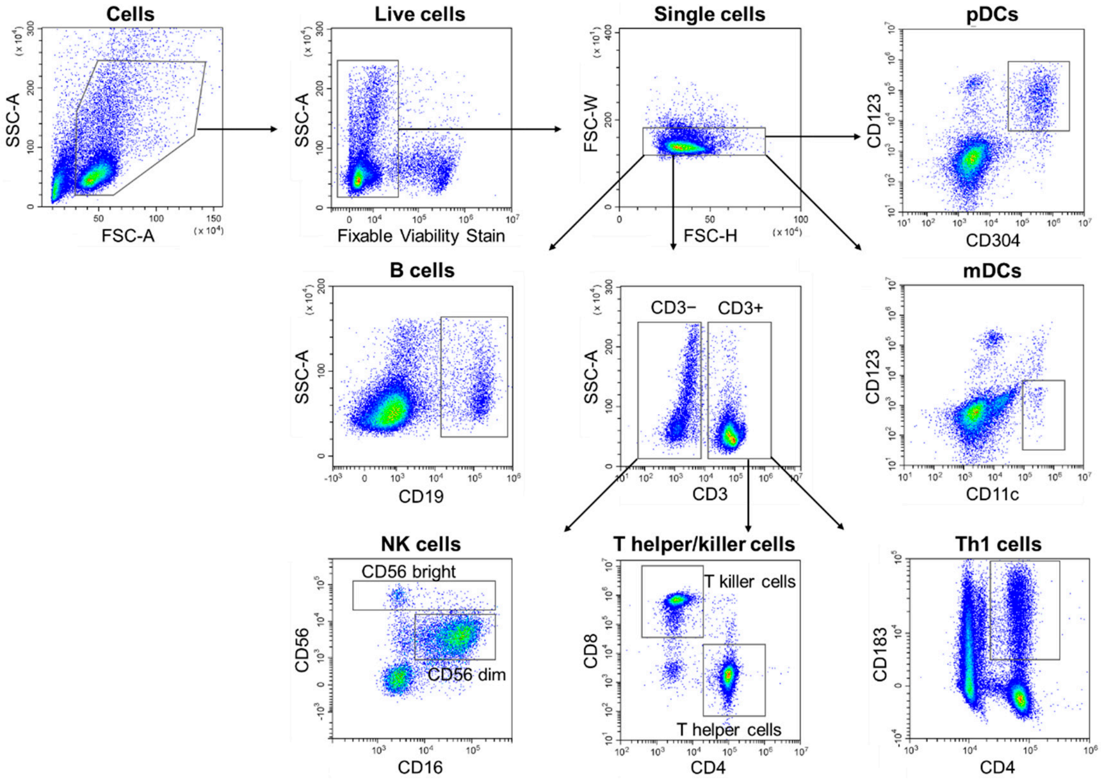


**Figure S1** Gating strategy to observe pDCs, mDCs, NK cells, Th1 cells, T helper cells, T killer cells and B cells in PBMCs.
